# Supplementary material for: Platinum recycling going green via induced surface potential alteration enabling fast and efficient dissolution
Source: Nat Commun. 2016 Oct 21;7:13164. doi: 10.1038/ncomms13164 (PMC5078734; doi:10.1038/ncomms13164)
Supplement: Supplementary Figures and Table — Supplementary Figures 1-7 and Supplementary Table 1. [file ncomms13164-s1.pdf]

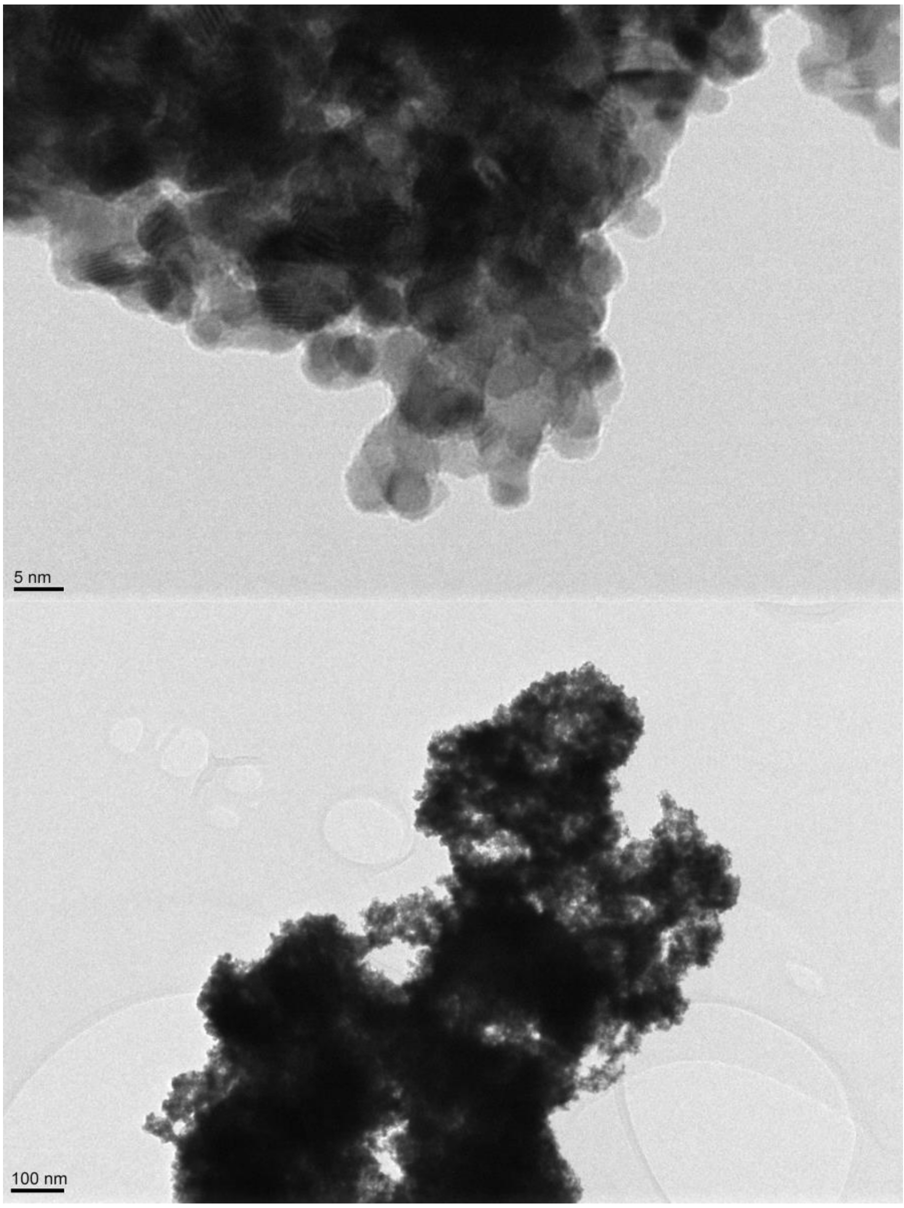

Supplementary Figure 1: TEM images of Pt-Black electrocatalysts supplied by Umicore.

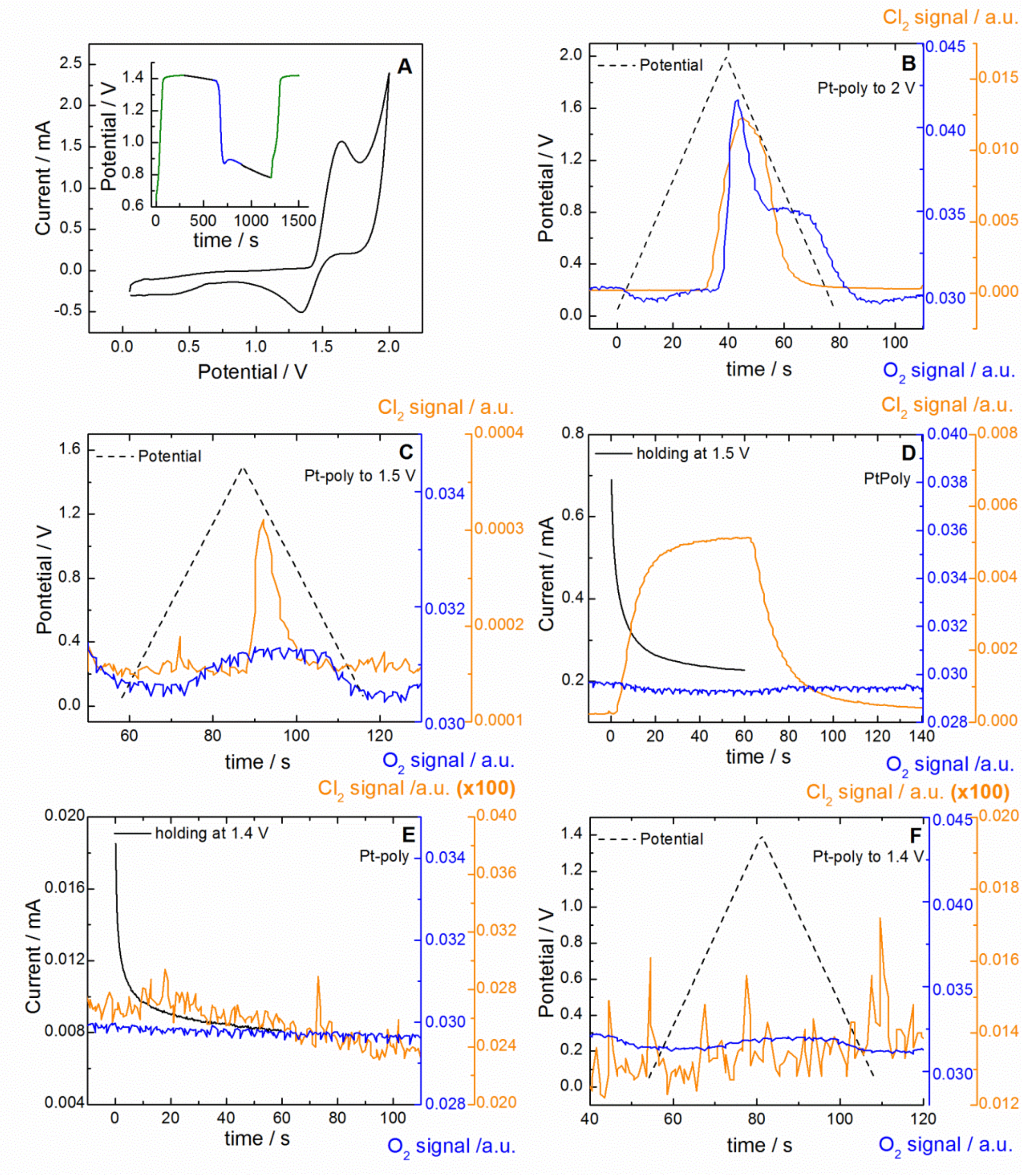

Supplementary Figure 2: (A) Cyclic voltammogram of Polycrystalline Pt in 0.1 M NaCl and 0.1 M $HClO_4$ and OCP profile upon 5 min ozone, 5 min Argon, 5 min CO, 5 min Argon and 5 min ozone (inset). Scanning Flow Cell (SFC) coupled to online electrochemical mass spectrometer (OLEMS) Cl_2 and O_2 responses after exposing Polycrystalline Pt in 0.1 M NaCl and 0.1 M $HClO_4$ to (B) 2 V cyclic voltammetry (50 mV/s), (C) 1.5 V cyclic voltammetry (50 mV/s), (D) 1.5 V potential hold, (E) 1.4 V cyclic voltammetry (50 mV/s) and (F) 1.4 V potential hold. All the potentials are referred to RHE. For Cl_2 and O_2 detection the fragments 35 and 32 were chosen respectively. It is clear from (A) that an upper potential around 1.4 V vs. RHE is triggered by ozone exposure and, that by cycling between 0.05 and 2.0 V vs. RHE there are two reactions taking place at 1.5 and 2.0 V vs. RHE. To understand the nature of these reactions the study continues with SFC-OLEMS. From Figure (B)-(F) we can conclude that at 2 and 1.5 V vs. RHE (although to a much lesser extent) there is Cl_2 and O_2 evolution. However at 1.4 V vs. RHE no gas evolution is observed. Therefore we can safely conclude two things: (i) chlorine evolution is the upper potential limiting reaction and (ii) chlorine evolution is minimal at the ozone OCP.

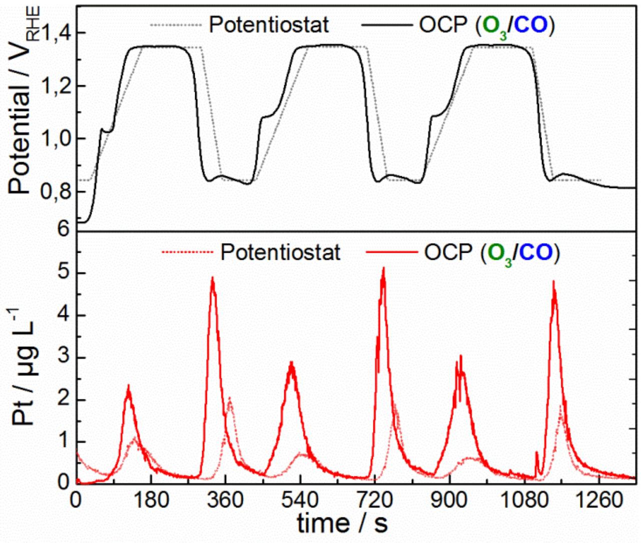

Supplementary Figure 3: SFC-ICPMS comparison of the Pt dissolution profiles for 3/3 minutes O_3/CO cycles and the potentiostatically simulated OCP variations.

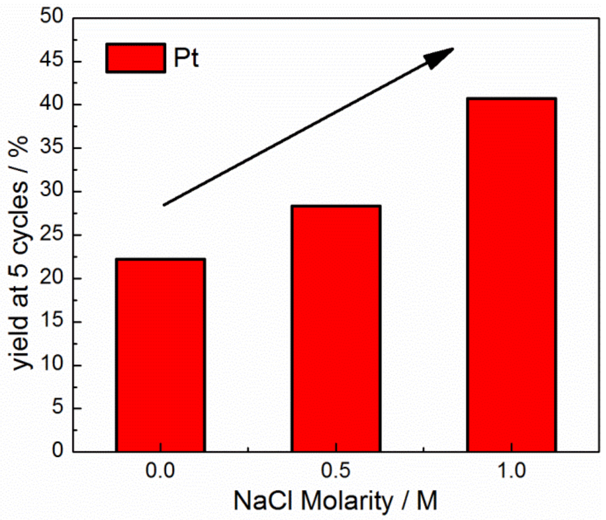

Supplementary Figure 4: Effect of NaCl molarity on the dissolution yield of Pt black after 5 cycles within the reactor.

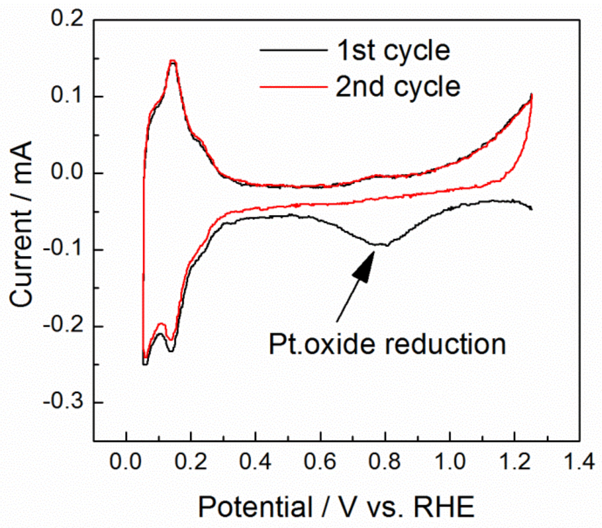

Supplementary Figure 5: Pt wire cyclic voltrammogram in the presence of 0.3 M HCl and 1 M NaCl. , i.e. a three-electrode assembly composed by a carbon rod, Pt wire and a reference electrode. The CO-induced potential perfectly fits to the electrochemical processes of oxide reduction and Pt oxidation. The reader can also see that the latter is a slow process since it takes 2 minutes at 1.23 V to produce a detectable Pt-oxide reduction peak, which is otherwise not visible if we cycle at 20 mV/s.

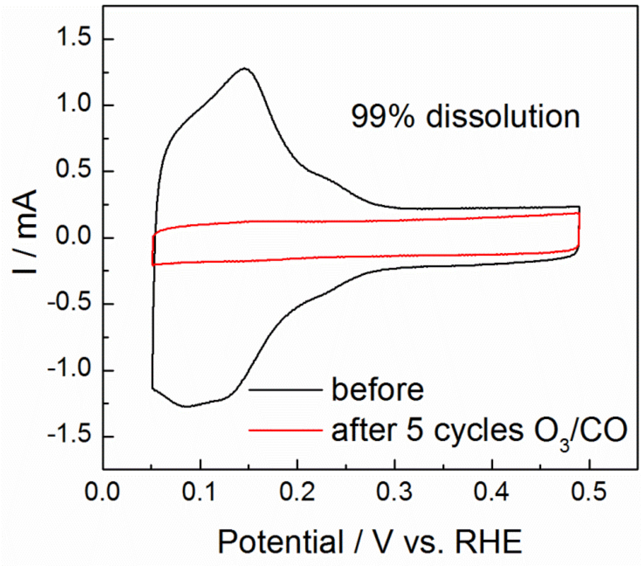

Supplementary Figure 6: Dissolution of Pt 3nm Tanaka catalyst in 100 ml $HClO_4$ and 10 l of 1M NaCl. Roughly 0.1 mg of the catalyst was deposited on a glassy carbon tip of an RDE and the dissolution progress was followed by monitoring the evolution of the H_{upd} area. Complete dissolution of the catalyst was achieved within 5 cycles (5/5 minutes O_3/CO).

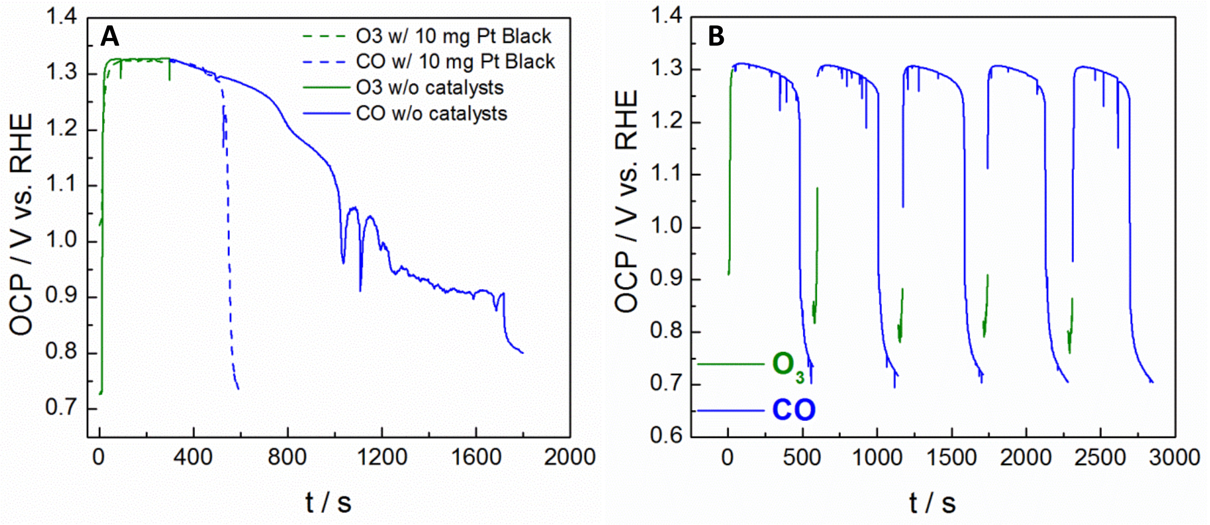

Supplementary Figure 7: (A) OCP measurement with and without 10 mg of Pt black in 100 ml 0.5 M HCl and 1 M NaCl solution. (B) *In operando* OCP measurement within the reactor (same electrolyte as above) in the presence of 1 g of catalytic convertor (for yield see Figure S8). The time of exposure of different gasses and experiments has to be adjusted due to the fact that the time to consume and/or displace the ozone by CO is dependent on the many parameters, one being amount of platinum in the suspension. Specifically, the presence of 10 mg Pt black in 100 mL shortens this time from 12 to 3.5 min. Thus, changing the amount of material to dissolve, the exposure to the gasses needs to be carefully adjusted to provide full oxidation and reduction.

Supplementary Table 1: Pt Black dissolution yields in 0.3 M HCl and 1 M NaCl after 20 cycles between 5 min ozone bubbling and 5 min CO bubbling. And Pt black dissolution yields after only ozone bubbling. 1 cycle is equal to 10 min.

cycles	% of Pt dissolved (O_3/CO)	σ (standard deviation)	cycles	% of Pt dissolved (O_3)
**5**	40,7	0,2	**5**	2,50311
**10**	68,0	1,7	**15**	4,02365
**15**	85,6	2,1	**30**	5,97609
**20**	98,6	0,5	**--**	
